# Diversity, mobility, and structural and functional evolution of group II introns carrying an unusual 3' extension

**DOI:** 10.1186/1756-0500-4-564

**Published:** 2011-12-28

**Authors:** Nicolas J Tourasse, Fredrik B Stabell, Anne-Brit Kolstø

**Affiliations:** 1 Laboratory for Microbial Dynamics (LaMDa), Department of Pharmaceutical Biosciences, University of Oslo, Oslo, Norway; 2 Institut de Biologie Physico-Chimique, UMR CNRS 7141, Université Pierre et Marie Curie, 13 rue Pierre et Marie Curie, 75005 Paris, France; 3 GeoKnowledge AS, Oslo, Norway

**Keywords:** Group II intron, Unusual extension, Evolution, pXO1-42, Plasmid, Mobility

## Abstract

**Background:**

Group II introns are widespread genetic elements endowed with a dual functionality. They are catalytic RNAs (ribozymes) that are able of self-splicing and they are also mobile retroelements that can invade genomic DNA. The group II intron RNA secondary structure is typically made up of six domains. However, a number of unusual group II introns carrying a unique extension of 53-56 nucleotides at the 3' end have been identified previously in bacteria of the *Bacillus cereus *group.

**Methods:**

In the present study, we conducted combined sequence comparisons and phylogenetic analyses of introns, host gene, plasmid and chromosome of host strains in order to gain insights into mobility, dispersal, and evolution of the unusual introns and their extension. We also performed in vitro mutational and kinetic experiments to investigate possible functional features related to the extension.

**Results:**

We report the identification of novel copies of group II introns carrying a 3' extension including the first two copies in bacteria not belonging to the *B. cereus *group, *Bacillus pseudofirmus *OF4 and *Bacillus sp*. 2_A_57_CT2, an uncharacterized species phylogenetically close to *B. firmus*. Interestingly, the *B. pseudofirmus *intron has a longer extension of 70 bases. From sequence comparisons and phylogenetic analyses, several possible separate events of mobility involving the atypical introns could be identified, including both retrohoming and retrotransposition events. In addition, identical extensions were found in introns that otherwise exhibit little sequence conservation in the rest of their structures, with the exception of the conserved and catalytically critical domains V and VI, suggesting either separate acquisition of the extra segment by different group II introns or a strong selection pressure acting on the extension. Furthermore, we show by in vitro splicing experiments that the 3' extension affects the splicing properties differently in introns belonging to separate evolutionary branches.

**Conclusions:**

Altogether this study provides additional insights into the structural and functional evolution of unusual introns harboring a 3' extension and lends further evidence that these introns are mobile with their extension.

## Background

Group II introns are genetic elements that are widespread in bacteria and in the organelles of eukaryotes. They are self-splicing catalytic RNAs (ribozymes) that remove themselves from precursor mRNA transcripts and ligate their flanking sequences (exons). Group II introns are also mobile retroelements which can invade genomic DNA sites [1-5]. Splicing can proceed through two major competing pathways, branchpoint or hydrolytic splicing [6-10]. Branchpoint splicing (or branching) involves two transesterification reactions, where the first reaction is initiated by nucleophilic attack on the 5' intron-exon junction by the 2' hydroxyl group of a specific bulged adenosine residue (the branchpoint) in domain VI near the 3' end of the intron. In the second reaction, the flanking exons are ligated and a branched intron lariat containing a 2'-5' linkage is released [1,2,5]. The hydrolytic pathway also consists of two steps; in the first step a water molecule acts as the nucleophile, and a linear intron is released after transesterification in the second step. Mobility occurs through reverse-splicing of the intron RNA into DNA and subsequent reverse-transcription by a multifunctional protein encoded by the intron (IEP, intron-encoded protein). Group II introns recognize and insert predominantly into cognate (homologous) intron-less sites in a process called retrohoming. Homing sites cover ~30 bp, and during splicing and reverse-splicing base-pairing interactions are made between a subset of these nucleotides (intron-binding sites, IBS, spanning positions -12 to +1 relative to the insertion site) and the complementary motifs in the intron RNA (exon-binding sites, EBS), while the distal regions are recognized by the IEP [1,2,4]. In addition, group II introns can insert into non-cognate (ectopic) sites that share partial similarity to the homing site in a process called retrotransposition, which occurs at a much lower frequency. In bacteria, group II introns are also often associated with other mobile genetic elements, such as insertion sequences and plasmids, that act as vectors for horizontal transfer [11-13].

The secondary structure of the group II intron RNA typically consists of six domains (numbered I to VI) that are linked by a network of tertiary interactions, and introns are classified based on structural features and IEP phylogeny [1,2,5,14-16]. However, we identified 15 copies of six unusual and different group II introns that carry a related 53/56-nucleotide (nt) extension at the 3' end [17-19]. All these introns were found in bacteria of the *Bacillus cereus *group, including *B. cereus*, *B. thuringiensis*, *B. mycoides*, and *B. pseudomycoides*. These bacterial species are genetically closely related and are known to harbor a range of mobile elements such as plasmids and introns [13,20-23]. Functional analysis demonstrated that the extra segment is part of the intron RNA molecule and affects the self-splicing reaction in vitro, and thus could be considered as a domain VII [17,18,24]. Phylogenetic analysis revealed that the unusual introns belong to two subgroups α and β within the bacterial B class [17,18]. In the present study we report the identification of the first introns with a 3' extension in bacterial species from outside the *B. cereus *group. Using the complete and diverse set of introns with an extra segment we conducted a detailed sequence and phylogenetic analysis of the introns together with their host genes and strains in order to gain insight into mobility, dispersal, and evolution of these elements and their domain VII. Functional studies were also carried out to investigate possible features related to the extension.

## Results and discussion

Sequence similarity searches of public sequence databases using BLASTN conducted in the present study revealed six additional group II introns carrying a 3' extension similar to those previously identified in refs [19,17], and [18] (Table 1). Interestingly, while all introns with a 3' extension known to date were found in closely related bacteria forming the *B. cereus *group, two of the newly discovered elements are encoded by strains of unrelated species, namely *Bacillus pseudofirmus *OF4 (previously classified as *B. firmus *OF4; [25]) and the uncharacterized *Bacillus sp*. 2_A_57_CT2. Phylogenetic analysis based on 16S ribosomal DNA sequences indicated that *B. sp*. 2_A_57_CT2 is close to *B. firmus *and that *B. sp*. 2_A_57_CT2, *B. pseudofirmus *OF4, and the *B. cereus *group are distantly related among the *Bacilli *(Additional file 1: Figure S1; [26]). These findings therefore extend the distribution of introns carrying an extra domain from the *B. cereus *group to the *Bacilli*.

**Table 1:** Currently identified group II introns carrying a domain VII

Intron^#,&^	Intron copy, Strain, Genbank accession number, Genomic coordinates	Intron's host gene predicted product	Phylogenetic subgroup within the B class	Reference
*B.c*.I4	a, *B. cereus *ATCC 10987 (plasmid pBc10987), AE017195, 35608-32766 b, *B. cereus *AH1271, ACMR01000217, 14976-17818 c, *B. cereus *AH1272, ACMS01000358, 3111-5953 d, *B. cereus *AH1273, ACMT01000367, 57936-55094	pXO1-70; hypothetical protein with DNA primase domain	α	[18,19]

*B.th*.I5	a, *B. thuringiensis kurstaki *BGSC 4D1/HD1, FM992108, 131-3040/*Contig365, 4502-7411^**$**^* b, *B. thuringiensis chinensis *CT-43 (plasmid pCT281), CP001910, 188422-191331	pXO1-08; hypothetical protein with two helicase domains	β	[17], This study

*B.th*.I6	a, *B. thuringiensis kurstaki *BGSC 4D1/HD1, FM992109, 370-3180/*Contig355, 4481-1671^**$**^* b, *B. thuringiensis kurstaki *BGSC 4D1/HD1, FM992110, 363-3174/*Contig362, 5366-2555^**$**^* c, *B. thuringiensis thuringiensis *ATCC 10792, ACNF01000191, 9655-12466 d, *B. thuringiensis thuringiensis *T01001, ACNA01000143, 13593-10782 e, *B. thuringiensis huazhongensis *BGSC 4BD1, ACNI01000192, 17280-20089 f, *B. thuringiensis chinensis *CT-43 (plasmid pCT281), CP001910, 139432-142243	pXO1-42; annotated as a protein belonging to the TraG/TraD family of plasmid proteins involved in bacterial conjugation, however, shows higher though weak homology to proteins of type IV secretion systems of the VirB/VirD family (see [27]).	β	[17,18], This study

*B.c*.I16	a, *B. cereus *Q1 (plasmid pBc239), CP000228, 228934-231746 b/c, *B. cereus *F65185, ACMO01000152, 2812-1	same as *B.th*.I6	β	[18]

*B.th*.I7	a, *B. thuringiensis kurstaki *BGSC 4D1/HD1, FM992111, 1064-3765/*Contig366, 3308-6009^**$**^* b, *B. thuringiensis kurstaki *BGSC 4D1/HD1, *Contig373, 943-1 + Contig381, 105890-104869^**$**^* c, *B. thuringiensis chinensis *CT-43 (plasmid pCT281), CP001910, 105318-102617	hypothetical protein (*B.th*.I7a and c) nucleoside transporter, NupC family (*B.th*.I7b)	α	[17], This study

B.my.I1	*B. mycoides *Rock1-4, ACMV01000578, 1-2843	not known due to missing sequence	β	[18]

*B.ps*.I1	*B. pseudomycoides *DSM 12442, ACMX01000035, 32580- 35423	intron inserted in non-coding region	β	[18]

*B.psf*.I1	*B. pseudofirmus *OF4 (plasmid pBpOF4-01), CP001879, 172131-169323	DNA primase	β	This study

*Ba.sp*.I2	*B. sp*. 2_A_57_CT2, ACWD01000076, 51450-54202	hypothetical protein	α	This study

**^#^**The entire nucleotide sequences (IEP-encoding ORF included) of *B.c*.I4a, b, c, and d are identical and inserted in the corresponding host gene. *B.th*.I5a and b differ at three nucleotide positions only. *B.th*.I6b, c, and d are identical to each other, while *B.th*.I6a and e are 98.4% and 99% identical to the former introns, respectively, and *B.th*.I6f differs from *B.th*.I6b, c, and d at a single nucleotide position. *B.c*.I16a and b/c are ~90% identical to the various *B.th*.I6 copies. All *B.th*.I6 and *B.c*.I16 copies are located in the same host gene. Due to missing sequence data, it could not be confirmed whether the *B.c*.I16 intron is present in one or two copies in the *B. cereus *F65185 strain. The entire nucleotide sequences of *B.th*.I7a and c are identical. Part of the IEP-encoding ORF sequence of *B.th*.I7b is missing from the genomic data, whereas the ribozyme sequence is complete and identical to that of *B.th*.I7a and c. The entire nucleotide sequences of B.my.I1 and *B.ps*.I1 are 96.5% identical

**^&^***B.th*.I6-like intron fragments were recently identified in two *B. thuringiensis *isolates from Mexico (Genbank accession numbers JF800177 and JF800178)

^**$**^Information in italics is based on sequence data from the Microgen website http://www.micro-gen.ouhsc.edu/b_thuring/b_thuringiensis_home.htm. Genome assembly from May 3, 2011

### Indirect evidence for mobility of the unusual introns with their extension

With respect to the phylogeny of the introns themselves, many of the introns with a 3' extension belong to the β subgroup within the bacterial B class [17,18], and in particular eight are highly similar to the *B.th*.I6 intron from *B. thuringiensis kurstaki *BGSC 4D1/HD1 (Table 1). The *B.th*.I6-like introns (*B.th*.I6 and *B.c*.I16) are all inserted in the same homing site within the pXO1-42 plasmid gene, and the two intron copies (*B.th*.I6a and b) found in *B. thuringiensis kurstaki *BGSC 4D1/HD1 were suggested to be the result of intron mobility [17,18]. Here, by combining all the available sequence data, supplemented by a PCR screen for pXO1-42, and by using the high sequence similarity between the introns and between the host genes, together with reconstructions of the phylogenetic relationships of the host genes and host strains, we attempted to detect further signs of intron mobility and to identify the events that have driven the dispersal of the unusual introns.

**Figure 1:**
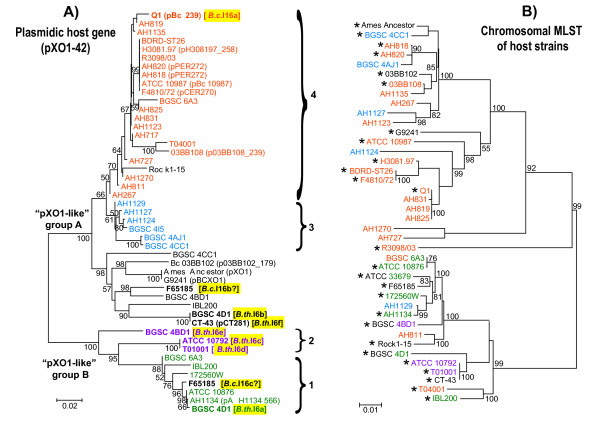
**Phylogenetic tree of pXO1-42 sequences from *B. cereus *group bacteria (A) and comparison with genetic relationships of the strains (B)**. Specific groups of strains have been colored to emphasize the incongruence between the two trees. In **A**), the tree was reconstructed from the nucleotide sequences of pXO1-42 using the Neighbor-Joining method applied to a pairwise distance matrix computed following Tamura's 3-parameter model. For strains with known plasmids, the plasmid name is given in parentheses. Strains whose pXO1-42 sequence contains an intron are shown in bold (intron name given in square brackets and yellow background; due to incomplete sequence data, it could not be confirmed whether strain F65185 carries one or two copies of *B.c*.I16). Numbered curly brackets indicate four pXO1-42 groups (strains labeled in different colors) sharing identical sequences around the intron's homing site, while remaining strains (in black) have variable sequences not belonging to these groups (sequences shown in Figure 2A). In **B**), the tree was extracted from a supertree of 1403 isolates based on multiple locus sequence typing (MLST) data of chromosomal housekeeping genes available in the SuperCAT database http://mlstoslo.uio.no/. No MLST data are available for strain BGSC 4I5. Strains whose genome has been completely sequenced are marked with asterisks. In **A**) and **B**) numbers next to branch nodes indicate statistical support values when > 50%. Scale bars are in average numbers of nucleotide substitutions per site. Origin and information about the strains can be found at the University of Oslo's typing website, http://mlstoslo.uio.no/. pXO1-42 was identified in four additional strains (AH1271, AH1272, AH1273, and AH717), however they were not included here because their genotyping data are conflicting (see [29])

**Figure 2:**
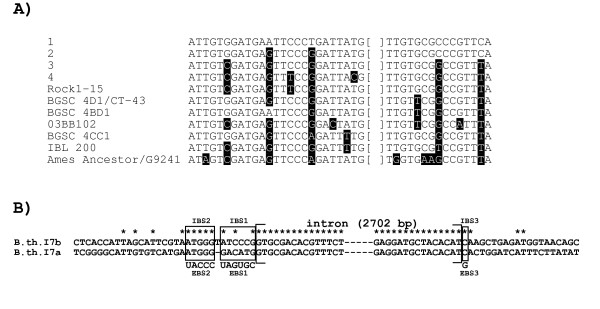
**A) Multiple alignment of *B.th*.I6 and *B.c*.I16 homing sites in pXO1-42 sequences**. The sequences shown span positions -25 to +15 around the intron insertion site (indicated by square brackets). Sequences numbered 1-4 correspond to the four groups shown in Figure 1A. Strains with different sequences not belonging to these groups (shown in black in Figure 1A) are individually listed by name (strain F65185 is not included due to incomplete sequence data). Nucleotide positions that differ relative to the top sequence are displayed in a black background. **B**). Insertion sites of the *B.th*.I7a and b intron copies in *B. thuringiensis kurstaki *BGSC 4D1/HD1. Sites that exhibit identical nucleotides in both sequences are indicated by asterisks. *B.th*.I7a and *B.th*.I7b have identical sequences and are inserted in plasmidic and chromosomal loci, respectively. Intron boundaries are delimited by brackets. The intron binding sites (IBS1, IBS2, and IBS3) in the exons are boxed and their complementary exon binding sites (EBS1, EBS2, and EBS3) in the intron are indicated underneath. The similarity between the insertion sites is weak overall, and is limited to the IBS2 motif, suggesting retrotransposition of *B.th*.I7 into ectopic sites

Altogether, the dataset included pXO1-42 sequences for 40 strains, including 12 sequenced large plasmids (180-560 kb) belonging to the "pXO1-like" family [28]. Phylogenetic analysis of the pXO1-42 sequences revealed interesting patterns. Firstly, the pXO1-42 phylogeny was largely inconsistent with the chromosomal MLST phylogeny (Figure 1), which indicates that there has been extensive horizontal transfer of "pXO1-like" plasmids disseminating this gene within the *B. cereus *group. Indeed, large plasmids are the main vectors of group II intron spread in this bacterial group [13]. Secondly, the pXO1-42 sequences were divided into two clusters separated by a long evolutionary branch and supported by a high statistical value (Figure 1A). This division is further supported by comparison of full plasmid or genome sequences, which confirmed that it corresponds to two groups of plasmids within the "pXO1-like" family, herein named "A" and "B" (Additional file 1: Figure S2). Thirdly, *B.th*.I6-like introns are distributed in both groups. Identical copies are present in distantly related pXO1-42 sequences, as exemplified by *B.th*.I6b and c/d and by *B.c*.I16a and b/c. This strongly suggests mobility of the individual introns rather than transfer of the whole locus containing host gene and intron (Figure 1A). A different example of intron mobility is given by the *B. cereus *Q1 strain which harbors *B.c*.I16a. This strain is part of a clonal complex including isolates AH819, AH825, and AH831 that has emerged recently in the MLST supertree (Figure 1B). The latter three isolates encode pXO1-42 sequences closely related to that of Q1 (with an identical homing site) but that are intron-less. This indicates therefore that an independent integration of *B.c*.I16a must have occurred in *B. cereus *Q1 quite recently in evolution. A last piece of evidence for mobility of *B.th*.I6-like introns is given by the fact that the sequence of the *B.th*.I6/*B.c*.I16 homing site also correlates with the phylogeny of the full pXO1-42 gene (Figures 1A and 2A). As this sequence interacts with the intron directly by basepairing, the presence of identical intron copies in different homing sequences strengthens the idea that this is the result of retrohoming by the introns.

Examination of the insertion sites of the *B.th*.I7a and b copies indicated that the *B.th*.I7 intron as well must be mobile with its extension. These two intron copies are inserted in different genes in *B. thuringiensis kurstaki *BGSC 4D1/HD1 (Table 1) and BLAST sequence similarity searches revealed that the genomic contigs containing *B.th*.I7a and *B.th*.I7b matched plasmidic and chromosomal *B. cereus *group sequences, respectively (data not shown). While the nucleotide sequences of the two intron copies are identical, the insertion sites exhibit little similarity, with the exception of the IBS2 motif (Figure 2B). This can be taken as evidence of retrotransposition of *B.th*.I7 into ectopic sites. This finding, added to the results presented above for the *B.th*.I6-like introns, shows that both retrohoming and retrotransposition of unusual group II introns in plasmid or chromosomal loci have occurred in *B. cereus *group genomes. However, no insight into the direction and precise history of these events could be inferred. *B.th*.I7 and the *B.th*.I6-like introns respectively belong to the α and β phylogenetic subgroup within the bacterial B class of group II introns [17,18], indicating that introns from both subgroups are (or have been) intrinsically mobile with their 3' extension.

### Identical 3' extensions in divergent introns: independent acquisition or high selection pressure?

In addition to providing evidence for mobility of the group II introns carrying an extra domain, detailed bioinformatic comparative analysis also revealed features that may be relevant to the structural evolution of these unusual introns. The first major feature is that divergent introns can share identical 3' extensions. This is case for the *B.th*.I6-like introns, where *B.th*.I6 from various *B. thuringiensis *strains and *B.c*.I16 from *B. cereus *Q1 and F65185 are 90% identical overall and have identical 54-nt extensions (Additional file 1: Figure S3A). Even more remarkable are the related B.my.I1 and *B.ps*.I1 introns from *B. mycoides *Rock1-4 and *B. pseudomycoides *DSM 12442, respectively, which also share a nearly identical extension with *B.th*.I6, but are more divergent overall, exhibiting only 60% nucleotide sequence identity to the *B. thuringiensis B.th*.I6 intron in domains I-VI (Additional file 1: Figure S3B; note that even though strain Rock1-4 is classified as *B. mycoides*, it actually belongs to the *B. pseudomycoides *lineage in the *B. cereus *group phylogenetic tree, see [18]). While the *B.th*.I6, *B.c*.I16, B.my.I1, and *B.ps*.I1 introns all belong to the β phylogenetic subgroup, there is also a case of an identical extension shared by introns of different subgroups: the 53-nt extension of *Ba.sp*.I2 from *B. sp*. A_2_57_CT2 (α subgroup) is identical to that of *B.th*.I5 from *B. thuringiensis *BGSC 4D1/HD1 and CT-43 (β subgroup; Additional file 1: Figure S3C). The presence of the same 3' extension in group II introns that otherwise show little conservation overall in the rest of their sequences could suggest that the extension may have been acquired independently by the different introns. Alternatively, as domain VII is important for the self-splicing reaction of the unusual introns [17]; see below), this could imply that there is a very strong selection pressure on the extension for structural and/or functional reasons, as is the case for domains V (the catalytic center of the ribozyme) and VI (containing the branchpoint), which, like domain VII, are highly conserved in sequence among introns of the B class (Additional file 1: Figure S3; [30]). One may also hypothesize that the conservation of domains V and VI could have favored homologous recombination events that could have mediated the transfer of domain VII between introns that are divergent in the rest of their sequences.

### A novel group II intron with a longer 3' extension of 70 nt in B. pseudofirmus

**Figure 3:**
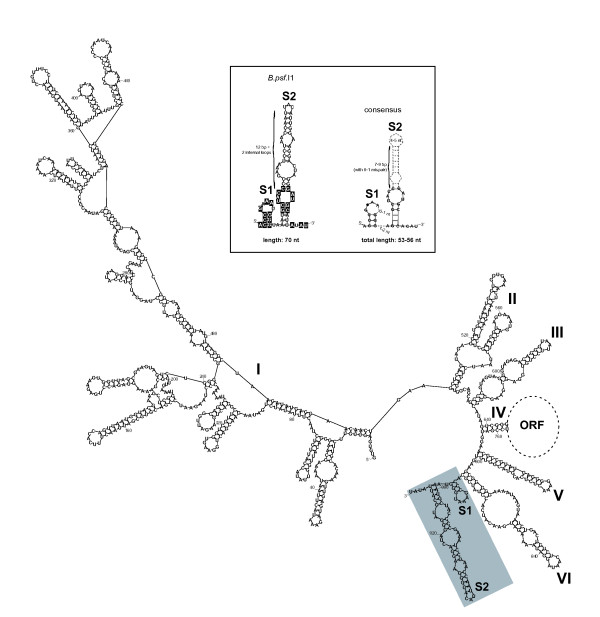
**Predicted secondary structure of the *B.psf*.I1 group II intron from *B. pseudofirmus *OF4 and comparison of its 3' extension with known domain VIIs**. Roman numerals (I to VI) indicate the six typical functional RNA domains. The extra 70-nt 3' segment is boxed in gray. ORF, intron-encoded multifunctional open reading frame. Numbering of residues does not include the ORF. The inset shows a comparison of the secondary structure of the 70-nt 3' extension of *B.psf*.I1 and the consensus structure of the 53/56-nt domain VII from the 20 other group II introns known to carry a 3' extension (see Table 1; consensus drawn as in ref. [18]). Sites in the *B.psf*.I1 extension that are identical to the consensus are drawn in a black background. Note the extended S2 stem in *B.psf*.I1

A second feature relating to the evolution of introns harboring a domain VII is given by the *B. pseudofirmus *OF4 intron, named *B.psf*.I1. While the 3' extra segment of all other unusual introns is 53-56 nucleotide long, the extension of *B.psf*.I1 spans 70 bases (Figure 3). It is predicted to fold into a 2-stem-loop structure (S1 and S2) similar to that of the *B. cereus *group elements, and, interestingly, the pattern of sequence and structure conservation between the extensions of *B.psf*.I1 and the *B. cereus *group introns is the same as that observed previously between the latter introns [17]. That is, first, the small stem S1 is highly conserved in sequence among all the unusual introns, including *B.psf*.I1. Second, while the S2 stem of *B.psf*.I1 is somewhat longer than that of the other unusual introns and is not conserved overall, the invariant internal loop representing a putative 11-nt tetraloop receptor motif present in *B. cereus *group introns [17] is identical in sequence in *B.psf*.I1, and is located at the same relative position within S2, i.e., 3 bp from the bottom of the stem (Figure 3). This conservation underscores that this motif must be important for intron structure and/or activity, as suggested by mutational analyses which showed that unpairing the G:C pair beneath the internal loop triggered a significant slowdown of the second splicing step [17]. Therefore, although being substantially longer, the 3' extension of the *B. pseudofirmus B.psf*.I1 intron shares all the features common to that of the other unusual introns and shows that domain VII itself can undergo evolutionary change while maintaining the key features.

### The 3' extension affects the splicing reaction differently in introns from the α and β subgroups

**Figure 4:**
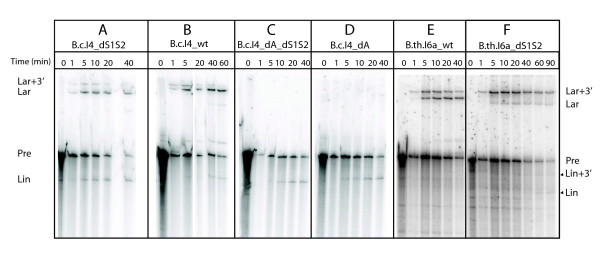
**In vitro self-splicing of *B.c*.I4 and *B.th*.I6a wild-type (WT) and mutant constructs in KCl-containing buffer**. (**A**) *B.c*.I4 deleted of the entire 3' extension (*B.c*.I4_dS1S2); (**B**) *B.c*.I4 WT; (**C**) *B.c*.I4 deleted of the entire 3' extension and the branchsite adenosine (*B.c*.I4_dA_dS1S2); (**D**) *B.c*.I4 deleted of the branchsite adenosine only (*B.c*.I4_dA); (**E**) *B.th*.I6a WT; and (**F**) *B.th*.I6a deleted of the entire 3' extension (*B.th*.I6a_dS1S2). Splicing was performed in 40 mM MOPS (pH 7.5), 500 mM KCl, and 100 mM MgCl_2 _at 47°C. Samples were separated on a 7 M urea 4% polyacrylamide gel. The various splicing products are labeled on the sides. The weak bands corresponding to the linear forms of *B.th*.I6a (panels E and F) are marked by arrowheads and were identified by size. "dS1S2" and "dA" refer to deletion of the entire 3' extension or the branchsite adenosine, respectively

**Figure 5:**
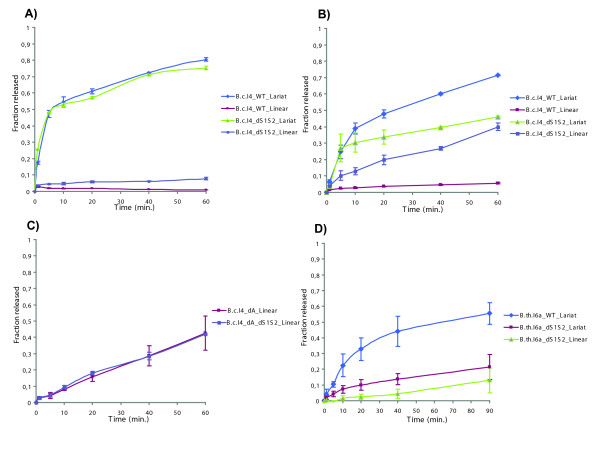
**Time-course analysis of in vitro self-splicing of *B.c*.I4 and *B.th*.I6a wild-type (WT) and mutant constructs**. (**A**) *B.c*.I4 WT and *B.c*.I4 deleted of the entire 3' extension (*B.c*.I4_dS1S2) spliced in (NH_4_)_2_SO_4 _buffer; (**B**) same constructs as in (A) spliced in KCl buffer; (**C**) *B.c*.I4 deleted of the branchsite adenosine only (*B.c*.I4_dA) or deleted of the branchsite adenosine and the entire 3' extension (*B.c*.I4_dA_dS1S2) spliced in KCl buffer; and (**D**) *B.th*.I6a WT and *B.th*.I6a deleted of the entire 3' extension (*B.th*.I6a_dS1S2) spliced in KCl buffer. Splicing was performed in 40 mM MOPS (pH 7.5), 100 mM MgCl_2 _and either 500 mM (NH_4_)_2_SO_4 _(panel A) or 500 mM KCl (panels B, C, and D) at 47°C. The relative fractions of released lariat intron were computed from the intensities of the radioactive bands using a phosphorimager. The values shown represent averages with standard deviations of one replicate from two different RNA preparations

The bioinformatic analyses presented above have given examples of the structural evolution of group II introns carrying a 3' extension. As RNA structure and function are intimately linked, in vitro mutational and kinetic analyses performed using introns belonging to the α and β phylogenetic subgroups revealed that these introns also evolved at the functional level. We showed previously that, while the *B.c*.I4 intron of *B. cereus *ATCC 10987 (α subgroup) has adapted to function with the 3' extra domain, the extension was not essential for splicing since the intron could splice nearly as efficiently as wildtype (WT) when the entire extension was deleted [24]. However, the deletion construct (*B.c*.I4_dS1S2) appeared to produce somewhat more linear form of the intron, suggesting more hydrolytic splicing without the 3' extension [17,24]. In the present study, we conducted time-course kinetic analyses of the self-splicing of the *B.c*.I4 WT and dS1S2 constructs. These analyses confirmed that the linear form is not the major product of the splicing reaction of WT *B.c*.I4, even in buffers containing KCl, which is known to promote the hydrolytic splicing pathway [8-10]. In KCl buffer the linear species only accounted for ~5% of the intron-containing products after 60 mins, whereas the fraction of free lariat was ~70% (Figures 4B and 5B). In comparison, splicing of the *B.c*.I4_dS1S2 construct produced a linear and lariat fraction of ~40% and ~45%, respectively, after the same time period (Figures 4A and 5B). These results demonstrate that the 3' extension has a clear impact on the balance between a hydrolytic or transesterification reaction in the first step of splicing. This may imply that the *B.c*.I4 intron with the 3' extension is either less prone to be hydrolyzed at the 5' splice site or is more efficient at branching. The bulged branchpoint adenosine in domain VI was therefore removed from the WT and dS1S2 constructs to investigate whether the increased hydrolysis of 5' splice site for dS1S2 could still be observed when there is no competition from this nucleophilic adenosine and the branching pathway. Comparison of the splicing of these two branchpoint-deleted mutant constructs, *B.c*.I4_dA and *B.c*.I4_dA_dS1S2, showed that the amount of free linear intron produced was very similar (Figures 4C and 4D and 5C). This suggests that the extension does not affect the rate of hydrolytic splicing directly. A more likely interpretation of the results may be that the 3' extension influences how efficiently domain VI and the branchpoint adenosine are positioned in the catalytic center with the 5' splice site.

Similar splicing experiments were conducted on the *B.th*.I6a intron from *B. thuringiensis kurstaki *BGSC 4D1/HD1 (β subgroup). Even though splicing of the *B.th*.I6a construct deleted of the full 54-nt 3' extension (*B.th*.I6a_dS1S2) also appeared to give an increased amount of free linear intron in KCl-containing buffer (~10% as opposed to 0% for WT; see Figures 4E and 4F and 5D), in sharp contrast to *B.c*.I4, splicing of *B.th*.I6a_dS1S2 in (NH_4_)_2_SO_4 _and KCl buffers showed a dramatic inhibition of the second splicing step. This was revealed by the accumulation of the first step intermediate "lariat + 3' exon" product compared to the *B.th*.I6a WT construct containing the extension (Figures 4E and 4F and 5D). The strong negative effect on the second splicing step observed for *B.th*.I6a is remarkable and shows that, unlike *B.c*.I4, *B.th*.I6a is dependent on the entire extension for efficient splicing. Together, this functional difference underlines that the two introns have adapted differently to the presence of a similar extension. *B.c*.I4 and *B.th*.I6a belong to separate evolutionary branches and exhibit sequence and structural differences that may be the basis for the observed splicing properties related to the extension.

## Conclusions

In conclusion, the sequence, phylogenetic, and experimental data presented in this study have revealed that the group II introns containing a 3' extension and their domain VII have had a dynamic relationship during evolution, both at the structural and functional levels. In addition, the data provided indirect, but clear, evidence that some of the unusual introns must be mobile with their extra segment. Altogether, this warrants structural and functional studies to better understand the structure-function relationship in group II introns carrying a domain VII, and to investigate the role and impact of the 3' extension in the mobility reaction.

## Methods

### Sequence homology searches

The nucleotide sequences of the previously identified *B. cereus *group introns carrying a 53/56-nt 3' extension [17-19] were used as queries to search the NCBI Genbank database [31] using BLASTN [32] for additional group II intron ribozymes having a similar extension. BLASTN was run with default parameters, except that the nucleotide match reward was set to 2 (-r 2). The genome sequence of *B. thuringiensis kurstaki *HD1 available at the Microgen website (Laboratory for Genomics and Bioinformatics, University of Oklahoma Health Sciences Center, Oklahoma City, USA; http://www.micro-gen.ouhsc.edu/b_thuring/b_thuringiensis_home.htm) was searched as well. Strain HD1 corresponds to strain BGSC 4D1, whose genome has also been sequenced by our laboratory and the Norwegian High-Throughput Sequencing Centre, University of Oslo, Norway (O. A. Økstad and L. Nederbragt, unpublished data). Introns were named following the nomenclature used in the Group II Intron Database [30].

The 86 completely sequenced *B. cereus *group strains publicly available in Genbank and Microgen at the time of analysis were screened for the *B.th*.I6 intron's host gene, pXO1-42, using BLAST. The BLAST search was conducted using the pXO1-42 sequence of the pXO1 plasmid of *B. anthracis *Ames Ancestor strain (locus tag GBAA_pXO1_0064) as query, and was performed both at the amino acid and nucleotide levels (run with default parameters, except E-value set to 0.01; -e 0.01).

An additional 36 isolates were screened by PCR, and positive products were sequenced. PCR was performed as previously described using the B.th.I6a_exon_left/right and B.th.I6b_exon_left/right oligonucleotide primer pairs [17]. Strains covering the phylogenetic diversity of the *B. cereus *group were selected for screening, including strains closely related to those harboring *B.th*.I6 and the *B.th*.I6-like *B.c*.I16 intron, based on the multiple locus sequence typing (MLST) supertree of chromosomal housekeeping genes available in the SuperCAT database at the University of Oslo's typing website ([33,29]; http://mlstoslo.uio.no/).

### Secondary structure predictions

The secondary structures of the *B.ps*.I1, *B.psf*.I1, and *Ba.sp*.I2 intron RNAs (IEP-encoding ORF removed) were computationally predicted by constrained folding using the MFOLD 3.1 package [34,35] following the consensus structures of group IIB (B class) introns [31,36]. That is, conserved and identifiable sequence motifs corresponding to the consensus structures were forced during the folding computation.

### Phylogenetic analyses

Homologous pXO1-42 nucleotide sequences were aligned using CLUSTALW 2 [37,38], followed by manual corrections done in SEAVIEW 4 [39,40]. A phylogenetic tree based on the multiple alignment was then reconstructed using the Neighbor-Joining method [41] applied to a matrix of pairwise distances between sequences. Evolutionary distances were computed according to Tamura's 3-parameter model [42], which takes into account multiple substitutions at a given site, differences between the rates of transitions and transversions, and G + C content bias. For strains with completely sequenced genomes or plasmids (marked with asterisks in Figure 1B) the full pXO1-42 sequence (~3.5 kb) was used, whereas a partial fragment of only ~400 bp that includes the homing site of *B.th*.I6-like introns was available for the strains that were screened by PCR. Thus, sites with gaps were removed in a pairwise manner when computing distances. Statistical support for branches in the tree was assessed by 1000 bootstrap replicates [43]. Phylogenetic analyses were done with MEGA 4.0.2 software [44].

The chromosomal phylogeny of the *B. cereus *group strains encoding the pXO1-42 gene was reconstructed using the MLST data available in the SuperCAT database ([33,29]; http://mlstoslo.uio.no/). The data included the nucleotide sequences of 7 to 26 chromosomal housekeeping genes, depending on the strain. According to information in culture collections, *B. thuringiensis kurstaki *BGSC 4D1 and ATCC 33679 should be the same strain and both correspond to strain HD1. However, typing studies revealed that the former two strains exhibit genotypic differences [29,45]. Thus, the sequence data for strains BGSC 4D1 and ATCC 33679 were included in the phylogeny. A supertree of 1403 *B. cereus *group isolates was reconstructed using the matrix representation by parsimony (MRP) technique as done in SuperCAT (see [33] for details), and the subtree containing the 40 pXO1-42-encoding strains was extracted from the supertree (pXO1-42 is also present in strains AH1271, AH1272, AH1273, and AH717, however these strains were not included in further analyses because their genotyping data are conflicting, see [29]). In order to obtain branch lengths that are proportional to numbers of nucleotide substitutions, branch lengths in the supertree were recomputed using PHYML 3.0 [46,47] and the Felsenstein-1984 nucleotide substitution model [48] supplemented with a gamma distribution (F84 + Γ). This model allows for unequal base frequencies, transition/transversion rate bias, and gamma-distributed substitution rate variation among sites. Statistical support for branches in the supertree was assessed by approximate likelihood ratio tests with Shimodaira-Hasegawa-like support values [33,46,49].

### Site-directed mutagenesis

Site-directed mutagenesis to generate intron constructs *B.c*.I4_dA, *B.c*.I4_dA_dS1S2, and *B.th*.I6a_dS1S2 was performed with Quikchange II (Stratagene) according to the manufacturer's instructions using two complementary oligonucleotides (of ~40 bases) containing the desired mutation(s) with either *B.c*.I4 or *B.th*.I6a ΔORF constructs as templates [17,24]. Primers are listed in Additional File 1: Table S4. Deletion of the 3' extension from *B.th*.I6a (*B.th*.I6a_dS1S2 construct) was performed in the same manner as done previously for *B.c*.I4 (*B.c*.I4_dS1S2 construct; [24]), i.e., by maintaining the last three nucleotides before the 3' splice site. All constructs were verified by sequencing.

### In vitro transcription

1 μg of plasmid construct was linearized by XhoI for transcription reactions with 30 U T7 RNA polymerase (Ambion) according to the manufacturer's instructions. Transcription and gel-purification of radiolabelled and unlabelled RNA were conducted as previously described [24].

### In vitro self-splicing of ribozyme

In vitro generated transcripts were denatured and refolded using a GenAmp 2700 PCR machine (Applied Biosystems), by incubating the transcripts in 10 mM MOPS, pH 7.5 at 90°C for 1 min, 75°C for 5 min, and then slow cooling to the splicing temperature of 47°C. Intron transcripts were spliced with 70000 cpm RNA or ~0.1 μg unlabelled transcripts in 40 mM MOPS, pH 7.5, 100 mM MgCl_2_, and either 500 mM (NH_4_)_2_SO_4 _or 500 mM KCl at 47°C. Reactions were initiated by adding pre-warmed splicing buffer to the transcript RNA giving a total reaction volume of 40 μl. At each time point of the time-course analysis, 2 μl were taken out, quenched with loading buffer (Ambion) and storing samples on dry ice. Samples were then heated to 95°C and cooled on ice, before being separated on a 7.5 M Urea 4% polyacrylamide gel. Gels were then vacuum dried, exposed, and analyzed using a Molecular Dynamics Storm 860 Phosphorimager.

For subsequent RT-PCR and sequencing of these splicing products, either unlabeled spliced transcripts, purified with Nucleotide purification kit (Qiagen), or labeled spliced transcript species, excised from gels, were used as templates.

For kinetic analysis, the intensities of the radioactive bands were quantified using the ImageQuant 5.0 software. The relative fractions of unspliced precursor and free lariat RNA were computed from the intensities of the radioactive bands of all intron-containing products.

## Competing interests

The authors declare that they have no competing interests.

## Authors' contributions

NJT and FBS jointly conceived the study and wrote the manuscript. NJT conducted the bioinformatic analyses. FBS performed the laboratory experiments. ABK supervised the study and contributed to the interpretation of the data. All authors have read and approved the current manuscript.

## Supplementary Material

Additional file 1 **Figure S1-S3 and Table S4**. The file includes three supplementary figures (S1-S3) along with the corresponding legends and associated references. The figures show respectively a phylogenetic tree of *Bacillus *species (Figure S1), comparisons of sequence homology between large plasmids (Figure S2), and drawings of the secondary structure of unusual group II introns (Figure S3). The file also includes a supplementary table (S4) listing the oligonucleotide primers used for the in vitro splicing experiments. Click here for file
