# Supplementary material for: Diversity, mobility, and structural and functional evolution of group II introns carrying an unusual 3' extension
Source: BMC Res Notes. 2011 Dec 28;4:564. doi: 10.1186/1756-0500-4-564 (PMC3261151; doi:10.1186/1756-0500-4-564)
Supplement: Additional file 1 — Figure S1-S3 and Table S4. The file includes three supplementary figures (S1-S3) along with the corresponding legends and associated references. The figures show respectively a phylogenetic tree of Bacillus species (Figure S1), comparisons of sequence homology between large plasmids (Figure S2), and drawings of the secondary structure of unusual group II introns (Figure S3). The file also includes a supplementary table (S4) listing the oligonucleotide primers used for the in vitro splicing experiments. [file 1756-0500-4-564-S1.PDF]

Supplementary Fig. S1

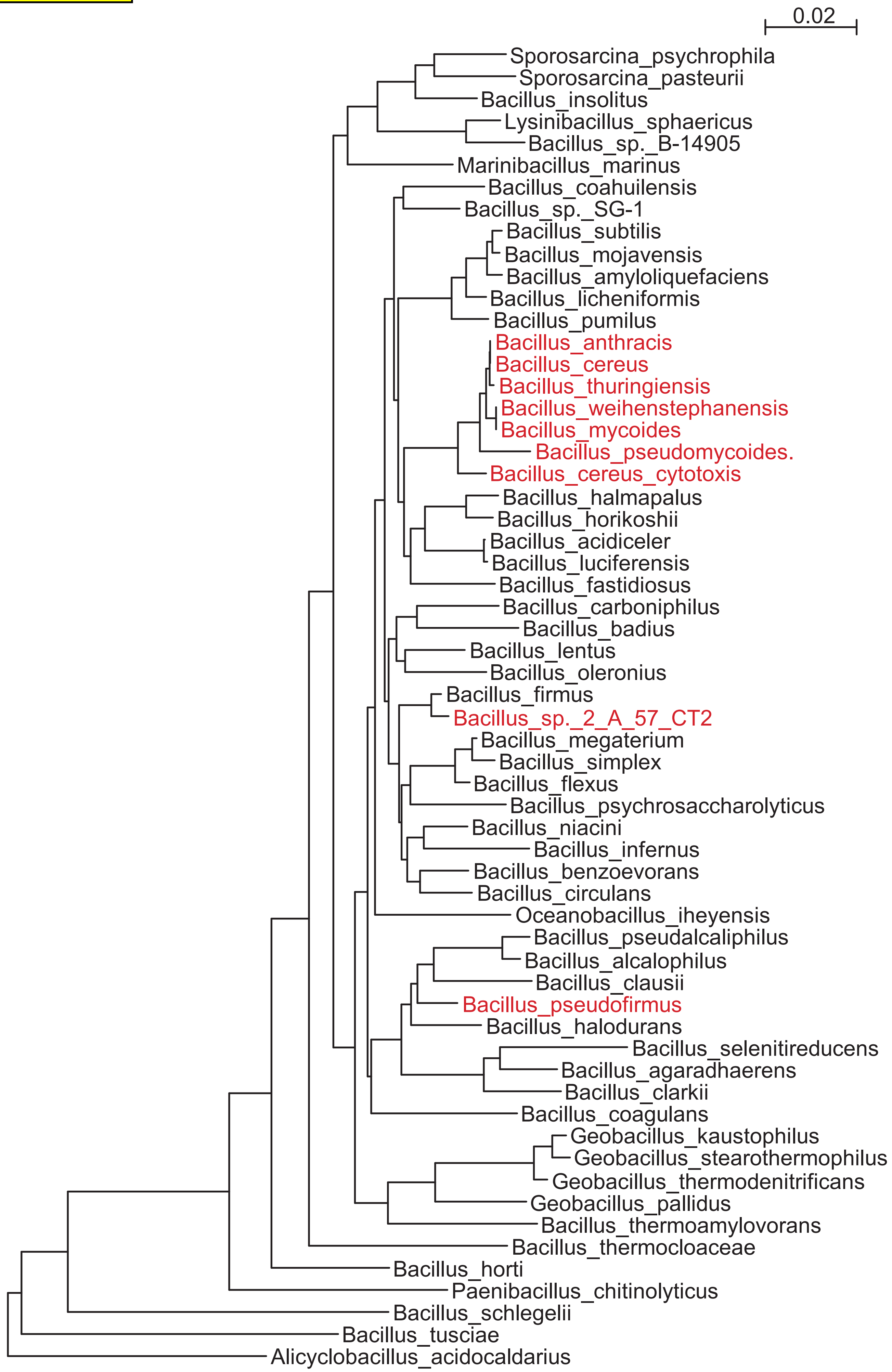

Supplementary Fig. S2

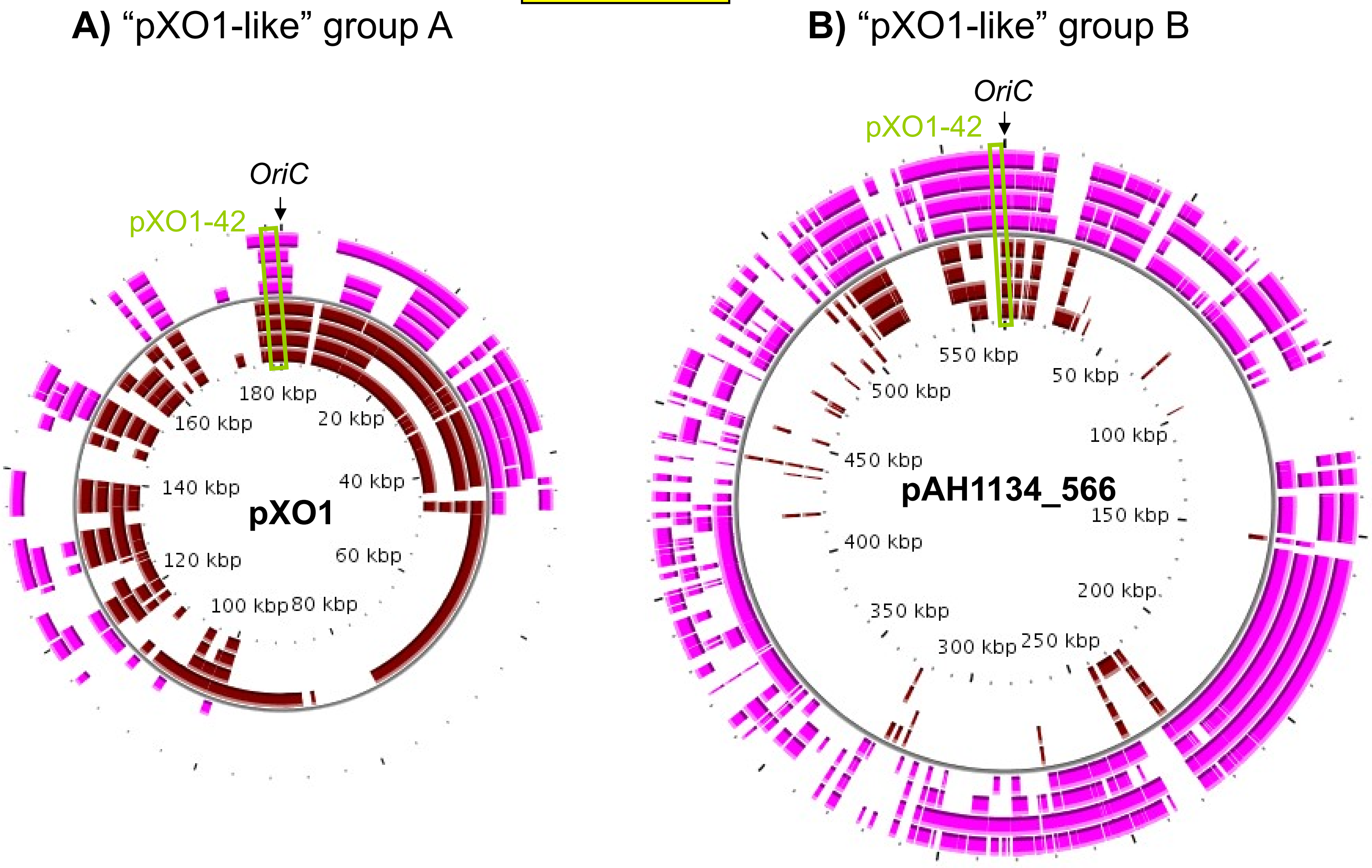

Supplementary Fig. S3A

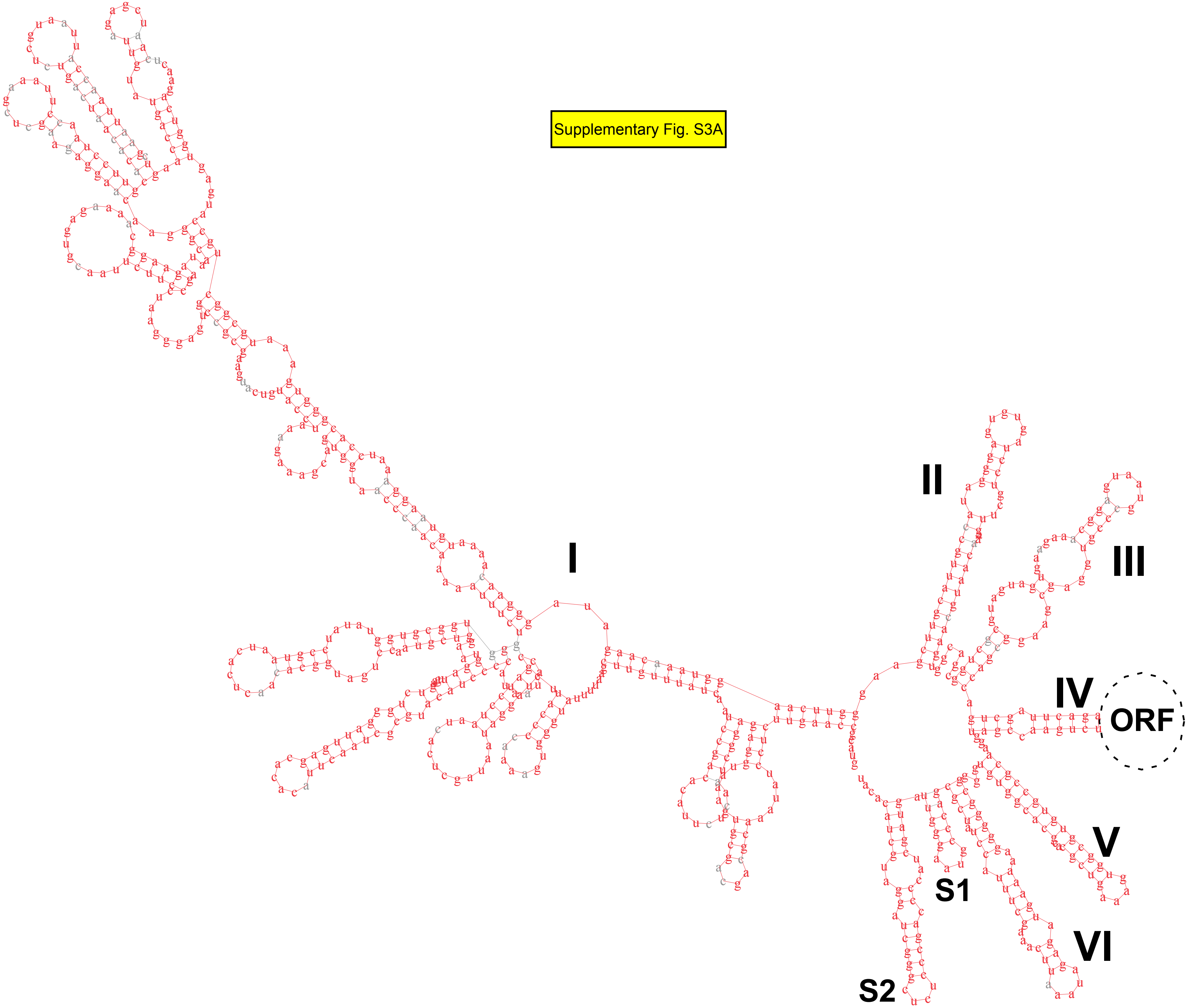

Supplementary Fig. S3B

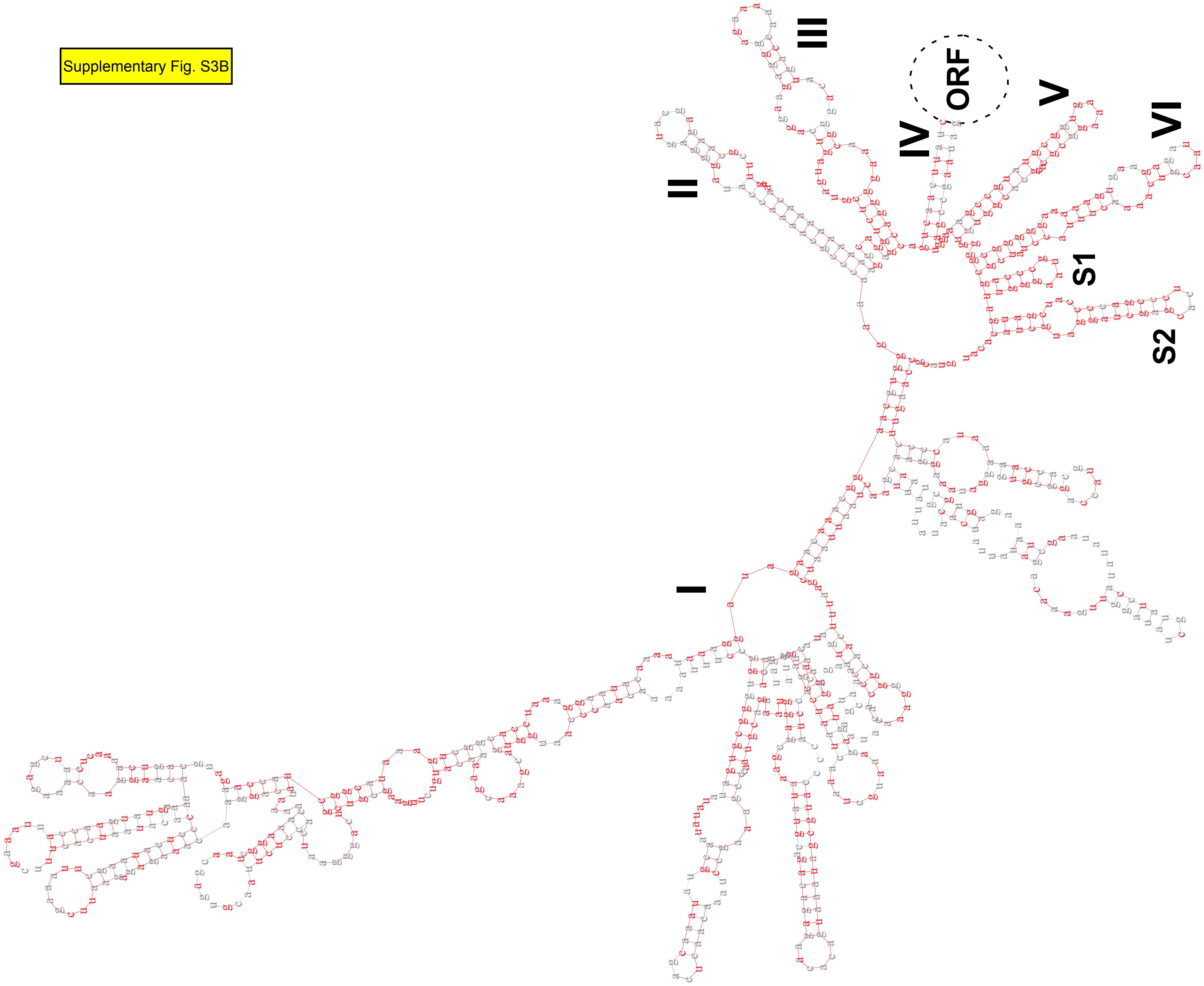

Supplementary Fig. S3C

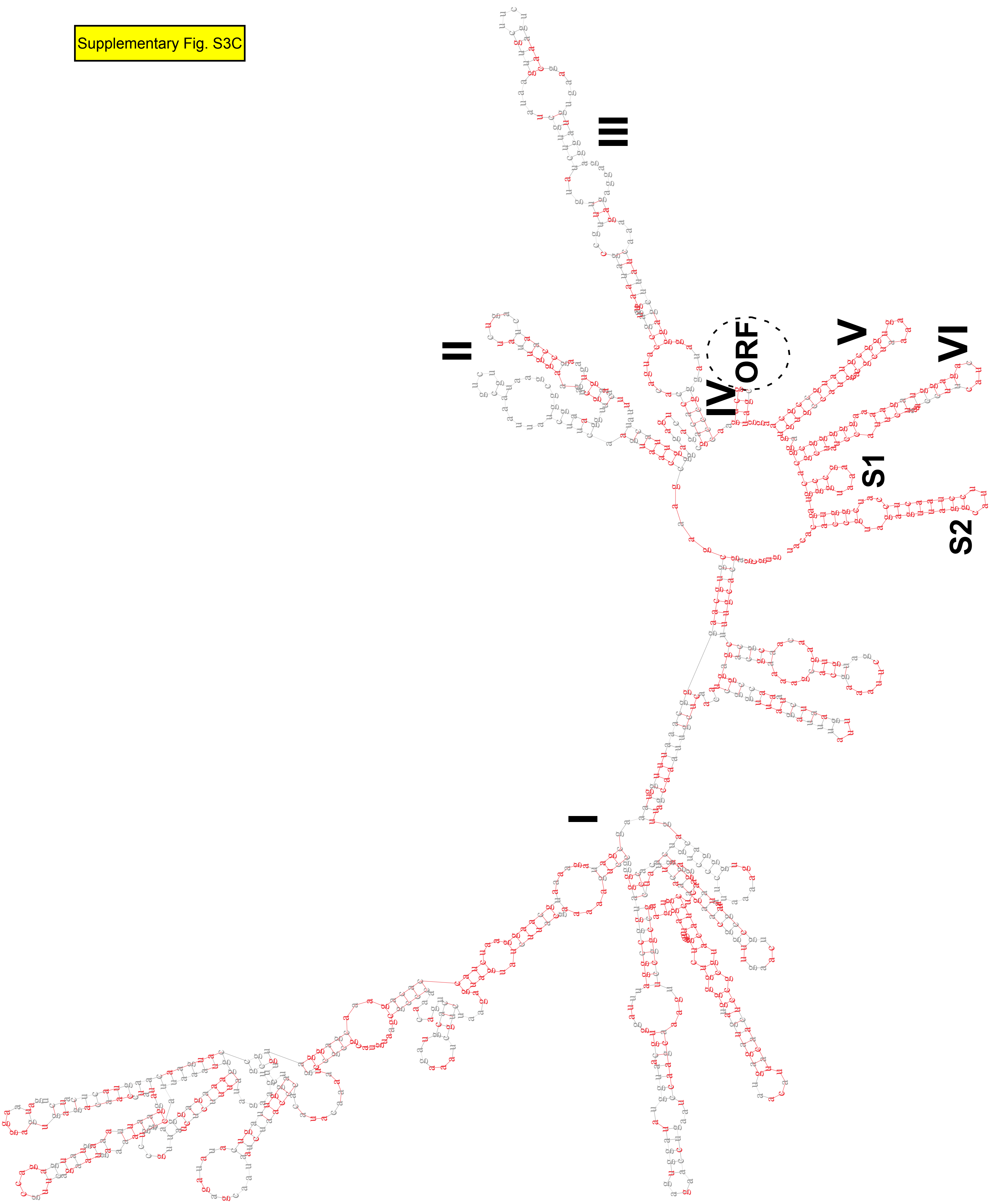

Legends to Supplementary Figures

Fig. S1. Relationships among a subset of 60 *Bacillus* species based on 16S ribosomal DNA (rDNA) sequences. A randomly chosen strain was used to represent each species. Species carrying group II introns with a 3' extension are highlighted in red. *Alicyclobacillus acidocaldarius* was used as an outgroup to root the tree. A multiple alignment of 16S rDNA sequences was downloaded from the Ribosomal Database Project (RDP) website (http://rdp.cme.msu.edu/) [1] and the 16S rDNA of *B. sp.* 2_A_57_CT2 (Genbank accession number ACWD01000095) was added to this alignment. After removing ambiguously aligned regions and all positions containing gaps (final alignment length of 1222 nt), a phylogenetic tree was reconstructed by means of the Neighbor-Joining method [2] applied to a matrix of pairwise distances between sequences, as described in [3]. Evolutionary distances were computed using the Tamura and Nei nucleotide substitution model [4] and MEGA 4.0.2 software [5]. The scale bar is in average number of nucleotide substitutions per site.

Fig. S2. Sequence homology between plasmids of groups A and B within the "pXO1-like" family. Sequence fragments of 500 bp or more exhibiting 80% or more nucleotide sequence identity are shown on circular representations of plasmids. Sequences of group A and B plasmids are drawn in brown and pink, respectively. In A, the pXO1 plasmid of *B. anthracis* Ames Ancestor was taken as the reference and was compared with the group A plasmids of *B. cereus* H3081.97, *B. cereus* Q1, *B. cereus* ATCC 10987, and *B. cereus* 03BB102, and the group B plasmids of *B. cereus* AH1134, *B. thuringiensis* ATCC 10792, *B. cereus* 172560W, and *B. thuringiensis* IBL 200 (from innermost to outermost circle). In B, the pAH1134_566 plasmid of *B. cereus* AH1134 was taken as the reference and was compared with the group A plasmids of *B. cereus* Q1, *B. cereus* ATCC 10987, *B. cereus* 03BB102, and *B. anthracis* Ames Ancestor, and the group B plasmids of *B. cereus* ATCC 10876, *B. thuringiensis* ATCC 10792, *B. cereus* 172560W, and *B. thuringiensis* IBL 200 (from innermost to outermost circle). Comparisons were based on a BLASTN search of the full sequence of pXO1 or

pAH1134_566 against the other *B. cereus* group plasmids or genomes (all default parameters except nucleotide match reward *r*=2 and E-value $e=10^{-5}$). Plasmids of group A are 180-260 kbp in length, while those of group B are >500 kbp. The circular representations are centered on the middle of the conserved origin of replication (*OriC*; see [6]) which was set as base 1 (original coordinate 55852 bp in pXO1 and 516386 bp in pAH1134_566). The location of the pXO1-42 gene is indicated by a green box. Images were generated using CGView [7].

Fig. S3. Comparison of the secondary structures of group II intron RNAs sharing an identical 53/54-nt 3' extension: (A) *B.th.*I6a from *B. thuringiensis* BGSC 4D1/HD1 and *B.c.*I16a from *B. cereus* Q1; (B) *B.th.*I6a from *B. thuringiensis* BGSC 4D1/HD1 and *B.ps.*I1 from *B. pseudomycoides* DSM 12442; (C) *B.th.*I5a from *B. thuringiensis* BGSC 4D1/HD1 and *Ba.sp.*I2 from *B. sp.* A_2_57_CT2. Each panel displays the consensus secondary structure generated from the alignment of the two intron structures computed using RNAforester [8, 9]. The most frequent base at each position is shown, with the base frequency indicated by grayscale (i.e., the lighter a base is drawn, the less frequent it is present in the individual structures of the four introns). Absolutely conserved bases or basepairs are drawn in red. Roman numerals (I to VI) indicate the six typical functional RNA domains. S1 and S2 are the two stem-loop structures making up the extra 53/54-nt 3' extension.

Supplementary references

1. Cole JR, Wang Q, Cardenas E, Fish J, Chai B, Farris RJ, Kulam-Syed-Mohideen AS, McGarrell DM, Marsh T, Garrity GM *et al*: **The Ribosomal Database Project: improved alignments and new tools for rRNA analysis**. *Nucleic Acids Res* 2009, **37**(Database issue):D141-145.
2. Saitou N, Nei M: **The neighbor-joining method: a new method for reconstructing phylogenetic trees**. *Mol Biol Evol* 1987, **4**(4):406-425.
3. Kolstø AB, Tourasse NJ, Økstad OA: **What sets *Bacillus anthracis* apart from other *Bacillus* species?** *Annu Rev Microbiol* 2009, **63**:451–476.
4. Tamura K, Nei M: **Estimation of the number of nucleotide substitutions in the control region of mitochondrial DNA in humans and chimpanzees**. *Mol Biol Evol* 1993,

10(3):512-526.

5. Tamura K, Dudley J, Nei M, Kumar S: **MEGA4: Molecular Evolutionary Genetics Analysis (MEGA) software version 4.0**. *Mol Biol Evol* 2007, **24**(8):1596-1599.
6. Rasko DA, Rosovitz MJ, Økstad OA, Fouts DE, Jiang L, Cer RZ, Kolstø AB, Gill SR, Ravel J: **Complete sequence analysis of novel plasmids from emetic and periodontal *Bacillus cereus* isolates reveals a common evolutionary history among the *B. cereus*-group plasmids, including *Bacillus anthracis* pXO1**. *J Bacteriol* 2007, **189**(1):52-64.
7. Stothard P, Wishart DS: **Circular genome visualization and exploration using CGView**. *Bioinformatics* 2005, **21**(4):537-539.
8. Höchsmann M, Töller T, Giegerich R, Kurtz S: **Local similarity in RNA secondary structures**. *Proceedings / IEEE Computer Society Bioinformatics Conference* 2003, **2**:159-168.
9. Höchsmann M, Voss B, Giegerich R: **Pure multiple RNA secondary structure alignments: a progressive profile approach**. *IEEE/ACM transactions on computational biology and bioinformatics / IEEE, ACM* 2004, **1**(1):53-62.

Supplementary Table S4: List of primers used for *in vitro* splicing analyses.

Primer name	Sequence[a]
B.c.I4_dA_dS1S2_sense	5'-gaaaaattggagataacatcaaagatttacct( )tcgca[ ]attttatgaaatgaag-3'
B.c.I4_dA_dS1S2_antisense	5'-cttcatttcataaaat[ ]tgcga( )aggtaaatctttgatgttatctccaatttttc-3'
B.c.I4_dA_sense	5'-attggagataacatcaaagatttacct( )tcgcacccgaaatg-3'
B.c.I4_dA_antisense	5'-catttcgggtgcga( )aggtaaatctttgatgttatctccaat-3'
BthI6a_dS1S2_sense	5'-gctttacctatcgcga[ ]atttgtgcgcccgttc-3'
BthI6a_dS1S2_antisense	5'-gaacgggcgcacaaat[ ]tcgcgataggtaaagc-3'

[a]The deletion of the branchpoint adenosine and the 3’ extension is indicated by parentheses and square brackets, respectively.
